# Microbial succession in response to pollutants in batch-enrichment culture

**DOI:** 10.1038/srep21791

**Published:** 2016-02-24

**Authors:** Shuo Jiao, Weimin Chen, Entao Wang, Junman Wang, Zhenshan Liu, Yining Li, Gehong Wei

**Affiliations:** 1 State Key Laboratory of Crop Stress Biology in Arid Areas, College of Life Sciences, Northwest A&F University, Yangling Shaanxi 712100, P. R. China; 2 Departamento de Microbiología, Escuela Nacional de Ciencias Biológicas, Instituto Politécnico Nacional, 11340 México, D.F., Mexico

## Abstract

As a global problem, environmental pollution is an important factor to shape the microbial communities. The elucidation of the succession of microbial communities in response to pollutants is essential for developing bioremediation procedures. In the present study, ten batches of soil-enrichment subcultures were subjected to four treatments: phenanthrene, n-octadecane, phenanthrene + n-octadecane, or phenanthrene + n-octadecane + CdCl_2_. Forty pollutant-degrading consortia, corresponding to each batch of the four treatments were obtained. High-throughput sequencing of the 16S rRNA gene revealed that the diversity, richness and evenness of the consortia decreased throughout the subculturing procedure. The well-known hydrocarbon degraders *Acinetobacter, Gordonia, Sphingobium, Sphingopyxis*, and *Castellaniella* and several other genera, including *Niabella* and *Naxibacter*, were detected in the enriched consortia. The predominant microbes varied and the microbial community in the consortia gradually changed during the successive subculturing depending on the treatment, indicating that the pollutants influenced the microbial successions. Comparison of the networks in the treatments indicated that organic pollutants and CdCl_2_ affected the co-occurrence patterns in enriched consortia. In conclusion, single environmental factors, such as the addition of nutrients or selection pressure, can shape microbial communities and partially explain the extensive differences in microbial community structures among diverse environments.

Community succession is defined as the change in the species composition and abundance of an ecological community over time^1^, and understanding the regular patterns of changes in community structure with time is a basic objective of ecological research^2^. In nature, the community in an ecosystem is shaped by the environmental factors, and ecological succession occurs when these environment factors are modified. Studies of succession have primarily focused on animal and plant communities, and few have focused on microbial communities^3^. Microbes are the main decomposers of organic materials, and changes in microbial community are often associated with functional capabilities^4^. Furthermore, the diversity of microorganisms, particularly prokaryotes, offers the potential for adaptation to various habitats, including environments severely contaminated with hydrocarbons and heavy metals. This broad adaptability has great value for the bioremediation of damaged ecosystems, and the characterization of microbial community succession in response to pollutions could provide important insight into ecosystem processes.

Environmental contamination is a global problem, and most of the contaminated sites are characterized by the presence of complex pollutants, including inorganic and organic compounds^5^. Common organic pollutants include hydrocarbons released from oil spills, and common inorganic pollutants include heavy metals generated by mining activities and metallurgical industries. Aliphatic alkanes and aromatic compounds (organic pollutants), the most important groups of petroleum hydrocarbons, often coexist with heavy metals (inorganic pollutants) in the contaminated environments^6^. Among the pollutants, n-octadecane, phenanthrene, pyrene, Cd and Pb are prevalent in the ecosystems and they are toxic to ecosystems and humans. The removal of organic pollutants in the presence of heavy metals via microbial remediation is thus a fundamental topic in applied microbiology^7^. While the detection of indigenous bacteria with the capacity of hydrocarbon utilization and metal-tolerant ability, included species within the genera *Alcanivorax*, *Bacillus*, *Gordonia*, *Dietzia*, and *Pseudomonas* etc., in the oil-contaminated soils^8^ has evidenced the potential of microbial remediation. Moreover, learning the key organisms in the procedures of bioremediation is relevant to the development of optimal *in situ* bioremediation strategies^9^. Most relevant studies have focused on the cultured bacteria^10^,^11^,^12^. However, these isolates might not be the dominant degraders for pollutants, and these mono- or multi-functional isolates would typically be unable to remediate the complex pollution. Consequently, attention has turned to microbial consortia for the degradation of pollutants because the degradation efficiency of these communities of organisms is higher than those of pure cultures^13^. For example, PAH-degrading microbial consortia enriched from tsunami sediments degraded PAH mixed with fluorene and phenanthrene nearly completely within ten days^14^. Several studies of microbial successions in environments polluted with phenol, toluene, and chlorinated aliphatic hydrocarbons^15^, heavy metals^16^ and petroleum^17^,^18^, have demonstrated that microbial communities change depending on the environmental conditions. These studies were conducted in complex environments, the differences in microbial community might be attributed to the changes in co-varying environmental factors. However, the interactions between the microbial community and the changes of environmental factors are still far from clear, although these relationships are conducive to explore the ecological function of microorganisms.

Recently, network analysis of taxon co-occurrence patterns has been proofed as an ideal method to get insight view into the structure of complex microbial communities and the interactions among microorganisms, such as commensalism, competition and predation^19^. This analysis has been successfully applied to explore the co-occurrence patterns between microbial communities in diverse environments including marine water^20^, soil^19^ and activated sludge^21^. However, the techniques employed in most studies of microbial community structures, such as clone library analysis, terminal restriction fragment length polymorphism (T-RFLP) and denaturing gradient gel electrophoresis (DGGE) targeting 16S ribosomal RNA (rRNA) genes, provide limited information due to the small number of sequences analyzed^22^. The recent combination of network analysis approaches with microbial datasets generated by high-throughput sequencing^23^ has been employed extensively to analyze microbial communities in the plant rhizosphere and surrounding soil^24^, deep sea sediment^25^, wastewater treatment system^26^, and human intestinal tract^27^. This method can detect rare species in samples and describe the overall microbial community diversity^28^, providing opportunities to investigate the microbial community succession during pollutant degradation.

In the present study, we conducted a metagenomic to estimate the changes of microbial community in the process of hydrocarbon degradation. We selected n-octadecane and phenanthrene as typical aliphatic alkanes and aromatic compounds that are prevalent in oil-contaminated soil, and cadmium (Cd), a potentially hazardous trace metal that is highly toxic to humans, animals, plants, and microorganisms. Mixtures of these compounds are becoming increasingly prevalent in ecosystems. To ensure that the succession was due to the pollutions, microcosms were used to provide a simple model for understanding the interactions among the environmental factors and the microbial community^29^. The response of the microbial community composition to various pollutants was examined via high-throughput sequencing of the 16S rRNA gene. The aim of the present study was to investigate the succession patterns of the microbial community in response to a distinct combination of pollutants. The results will be valuable for estimating the effects of relevant pollutants on microbial communities in nature and guiding bioremediation practices.

## Results

### Degradation of pollutants by various consortia

In this study, forty enriched pollutant-degrading consortia were obtained, corresponding to the ten stages of subculture with the following four treatments: phenanthrene (PHE), n-octadecane (C18), phenanthrene + n-octadecane (PC), and phenanthrene + n-octadecane + CdCl_2_ (PCC). These consortia exhibited a high efficiency of pollutant degradation (details available as Supplementary Fig. S1 and Table S1): 87.5%–100% with an average of 96.6–99.7% for n-octadecane in the C18, PC and PCC treatments; and 29.5–95.8% with an average of 74.5–81.4% for phenanthrene in the PHE, PC and PCC treatments. In PC, the efficiency with which n-octadecane was degraded was unchanged, but the phenanthrene degradation was decreased compared with in PHE. In PCC (containing CdCl_2_), the biodegradation of both n-octadecane and phenanthrene was significantly decreased compared with in the other treatments.

### Bacterial composition of the consortia

After the quality filtering and the removal of chimeric sequences, the entire sequencing data set containing 1,249,221 sequences was obtained from the forty consortia and the original soil samples. The average number of sequences per sample (n = 41) was 30,469 (max = 63,878, min = 15,006, SD = 11,429). The total OTU number was 29,014, defined at 97% sequence similarity (Table 1). Among these OTUs, 90.53% (26,267 OTUs) were assigned to 38 phyla, 92 classes, 155 orders, 236 families and 694 genera (Supplementary Table S2). The original soil contained the highest number of OTUs and microbial taxa compared with the treatment consortia. At the phylum level, Proteobacteria and Bacteroidetes were the predominant groups in each treatment and the original soil, with relative average abundances of 56.28% and 34.66%, respectively. The abundance of Proteobacteria was greater than that of Bacteroidetes in the original soil (ratio of these two phyla, 55.30/23.10), PHE (63.33/29.51), PC (51.82/40.43) and PCC (72.86/24.02), whereas the opposite relationship was observed in C18 (37.19/45.86). Greater differences were observed at the class level among the distinct consortia (Supplementary Fig. S2). Betaproteobacteria was the dominant group in original soil and PC, with relative average abundances of 21.70% and 29.03%, respectively; Gammaproteobacteria was most abundant in PCC, at 47.38%; Alphaproteobacteria and Betaproteobacteria were dominant in PHE consortia; and Sphingobacteriia was prominent in C18.

### Pollutant effects on microbial patterns

**Figure 1:**
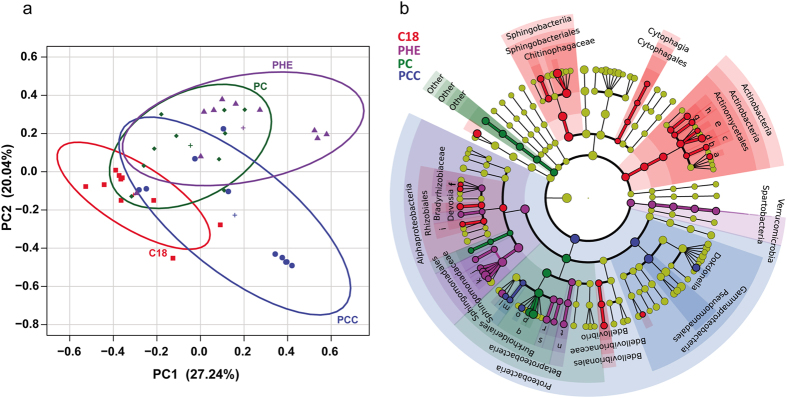
Distinct microbial patterns of the different treatments. (**a**) Weighted UniFrac distance principal coordinate analysis (PCoA) of microbial communities among the forty consortia in the four treatments. 80% confidence ellipses are shown around each treatment group. (**b**) LDA effect size taxonomic cladogram comparing all consortia categorized according to treatments. Significantly discriminant taxon nodes are colored, and branch areas are shaded according to the highest-ranked variety for that taxon. The yellow nodes correspond to taxa that were not significantly differentially represented between treatment groups. Highly abundant and selected taxa are indicated: a, *Microbarterium*; b, *Nakamurella*; c, Nakamurellaceae; d, *Gordonia*; e, Nodcardiaceae; f, *Nitrobacter*; g, *Aeromicrobium*; h, Nocardiodidaceae; i, Phyllobacteriaceae; j, *Novosphingobium*; k, *Sphingobium*; l, Pigmentiphaga; m, *Aquabacterium*; n, Nitrosomonadaceae; o, *Delftia*; p, Hydrogenophaga; q, Comamonadaceae; r, *Naxibacter*; s, Oxalobaceraceae; t, *Nitrosomonas*. For the complete list of discriminate taxa and ranks used to generate this cladogram, see Dataset S1.

Principal coordinate analysis (PCoA) (Fig. 1a) revealed that the bacterial community structure varied across treatments, with significant differences in bacterial diversity at the species (Bray-Curtis R_ANOSIM_ = 0.3305, *P* < 0.001; R^2^_ADONIS_ = 0.2265, *P* < 0.001) and genetic levels (Weighted UniFrac R_ANOSIM_ = 0.3048, *P* < 0.001; R^2^_ADONIS_ = 0.2828, *P* < 0.001).

Significant taxonomic differences between treatments were examined using LDA (least discriminant analysis) effect size (Lefse) based on the 108 main taxa (relative abundance >1%). The resulting significant taxa were used to generate taxonomic cladogram illustrating the differences among treatments (Fig. 1b). The classes Actinobacteria, Sphingobacteria and Cytophagia and the genera *Acinetobacter, Gordonia*, *Devosia, Bdellovibrio, Microbacterium, Aeromicrobium, Aquamicrobium, Nitrobacter* and *Nakamurella* were abundant in C18. In PHE treatment, the significantly abundant taxa were Alphaproteobacteria and Spartobacteria at the class level; Bradyrhizobiaceae and Oxalobacteraceae at the family level; and *Sphingobium, Sphingomonas, Novosphingobium, Naxibacter* and *Nitrosomonas* at the genus level. The phyla Betaproteobacteria, orders Rhodospirillales and Burkholderiales, families Comamonadaceae and Acetobacteraceae and genera *Hydrogenophaga* were significantly abundant in the consortia of PC. In PCC, the phyla Gammaproteobacteria, the orders Pseudomonadales, and the genera *Delftia*, *Dokdonella*, *Pigmentiphaga*, and *Aquabacterium* were abundant.

The selection of main taxa explaining the strongest variation between the treatments by the random forest (RF) supervised-learning classification model presented low OOB error rate 0.20 ± 0.12, indicating the model was adequate. The first 50 important taxonomic features for treatment prediction in these models were visualized using a heatmap (See detail in Supplementary Fig. S3), and they were mainly consistent with the significant taxa identified by Lefse (listed in the Supplementary Table S3), confirming the stability of the observations.

### Microbial community succession

**Figure 2:**
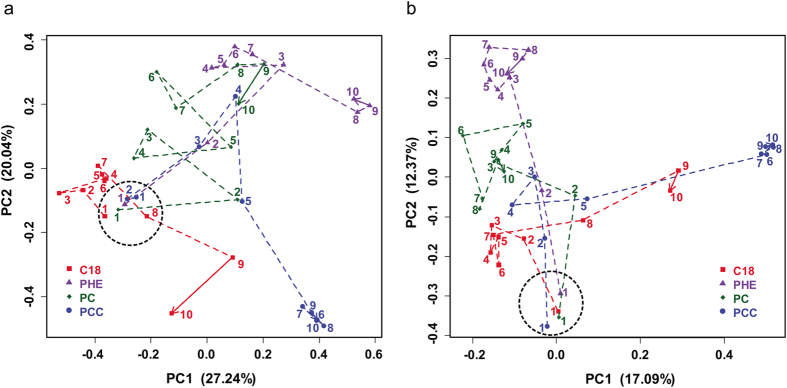
Microbial community succession. Weighted UniFrac (**a**) and Bray-Curtis (**b**) distance principal coordinate analysis (PCoA) of microbial communities among the forty consortia in the four treatments. The numbers accompanying the symbols represent the stages of enrichment culture. The directional development of the communities is indicated with arrows.

In the OTU-based analyses, the alpha diversity, including OTU richness, Chao1 richness, Shannon’s index and Pielou’s evenness, significantly decreased from the beginning to the later stages, as determined by linear regressions (Supplementary Fig. S4). PCoA based on distances of beta diversity distance revealed that the community structures were similar at the initial stage in the consortia of the different treatments and became increasingly dissimilar during the process of enrichment, and the final treatment-specific consortia were differentiated (Fig. 2). Plotting the dissimilarity in community composition between the first subculture and other subculture stages for each of the treatments (Supplementary Fig. S5a and b) revealed that the dissimilarities rapidly increased to a plateau of 80% from the third subculture (30 d) for all treatments. The dissimilarities between two adjacent subcultures within each treatment were decreased throughout the subculture procedure (Supplementary Fig. S5c and d), indicating that the consortium community became stable with the process of enrichment culture.

**Figure 3:**
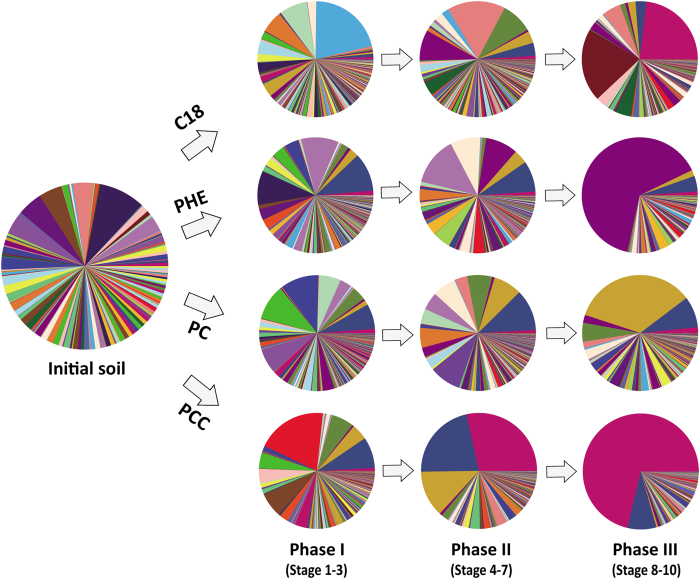
Pie charts of microbial composition at the OTU level for the 500 most abundant OTUs in the initial soil and the enriched consortia of the three phases of different treatments.

To investigate the change patterns in microbial succession, we divided the ten stages into three phases: phase I (the initial 1^st^–3^rd^ subcultures), phase II (the intermediate 4^th^–7^th^ subcultures) and phase III (the final 8^th^–10^th^ subcultures). The differences in microbial composition among the different treatments in each phase tested with ANOSIM and ADONIS are presented in Table 2. In phase I, the microbial community compositions did not differ significantly among the treatments (*P* > 0.1). By contrast, in phase II and III, significant differences were observed; the differences among the treatments were larger in phase III than in phase II. PCoA analysis revealed that the confidence ellipses of four treatments consortia enlarged gradually from phase I to phase III, suggesting that the differences in community structure increased as enrichment progressed (Supplementary Fig. S6). These differences were also confirmed by the microbial compositions estimated from the top 500 most abundant OTUs (Fig. 3). Initially, the relative abundances of these OTUs were primarily homogeneous (Pielou’s evenness = 0.827). The addition of pollutants remarkably changed the relative abundances of these OTUs, even in the first phase. After ten stages, pollutant-specific degraders were enriched from low abundance, and the microbial communities were stabilized. For example, when C18 was added, the relative abundance of Pseudomonadaceae increased from less than 1% in phase I to 20.54% in phase III. By contrary, Planctomycetaceae and Chitinophagaceae decreased. In addition, OTUs belonging to *Sphingobium*, Flavobacteriaceae and Pseudomonadaceae were predominant, accounting for nearly 60% in PHE, 30.41% in PC, and 66.41% in PCC in the final phases.

The Venn diagram of the relative abundances of the first 20 OTUs within the initial soil and the phases III consortia (Supplementary Fig. S7) revealed that no OTUs in the initial soil were detected in the enriched consortia, except a single overlapping OTU in PCC. Among the 20 most abundant OTUs, 7 to 9 were treatment specific, and 5 OTUs belonging to Pseudomonadaceae, Comamonadaceae, Flavobacteriaceae, Xanthomonadaceae and Sphingomonadaceae were shared by all the enriched consortia. The remaining OTUs were shared by some of the different treatments, in which PHE-PC and C18-PC had the largest overlaps with 10 OTUs.

### Comparison of co-occurrence networks among treatments

**Figure 4:**
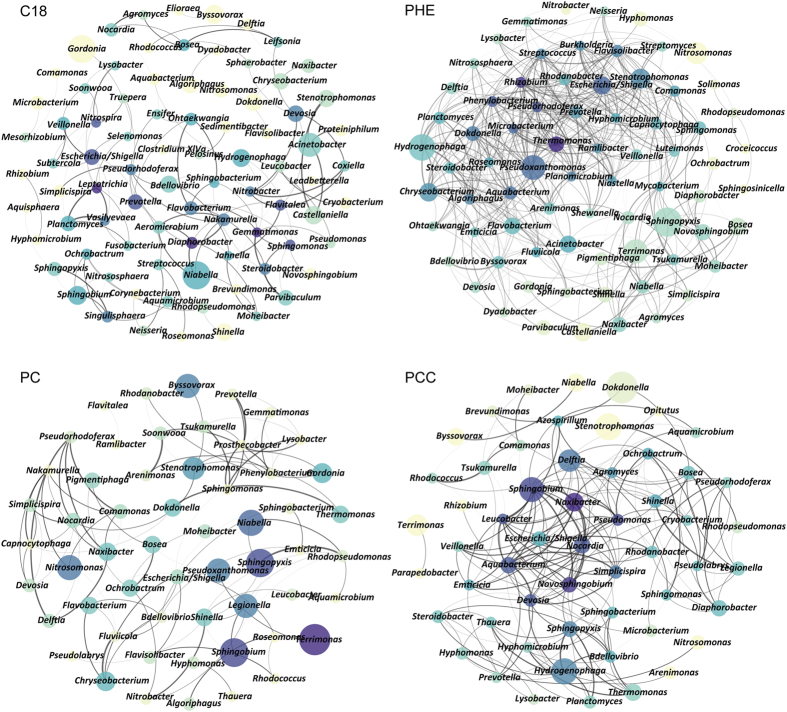
Network of co-occurring bacterial genera based on correlation analysis. A connection indicates a strong (Spearman’s ρ > 0.6) and significant (*P* < 0.01) correlation. The size of each node is proportional to the relative abundance; the color shade from dark-blue to yellow of each node is proportional to the number of connections (degree) from large to small, and the thickness of each connection between two nodes (edge) is proportional to the value of Spearman’s correlation coefficients.

The co-occurrence networks and the related topological properties for each treatment were presented in Fig. 4 and Table 3. The edge number in the obtained networks that present the correlations among the members in the communities varied between 513 for PHE and 95 for PC. The microbial community of PHE exhibited the highest node connectivity, with an average degree of 13.865, whereas that of C18 was lowest, with an average degree of 2.833. The average path length ranged from 2.695 to 5.620 edges, indicating that the average network distance was variable among all pairs of nodes. Furthermore, the highest (0.190) and the lowest (0.034) densities of the networks were also found in PHE consortia and C18 consortia, respectively, consisting with the clustering coefficient or the degree to which nodes tend to cluster together. In general, the PHE consortia hold more complex and compact correlation than PCC, followed by PC and C18.

## Discussion

Aiming at revealing the ecological effects of different contaminants and combinations of contaminants, we conducted a meta-analysis of the microbial community structure during an enrichment procedure.

Firstly, we observed that the organic pollutants played a role in selection of microbial degraders as substrates and as environmental factors. Among the pollutants used in the present study, only the organic pollutants n-octadecane and phenanthrene can be used as energy and carbon sources by chemoheterotrophs in the microcosms; therefore, all the bacteria in the consortia should be related to the degradation of these two compounds, as evidenced by the rates of removal of n-octadecane and phenanthrene in the microcosms (Supplementary Fig. S1). The higher removal rates of n-octadecane are reasonable because this straight-chain alkane is easier to utilize than phenanthrene (a polycyclic aromatic hydrocarbon) by the microorganisms. The community structure analyses of the consortia clearly revealed variations of the composition and relative abundance of the phylogenetic groups among the four treatments (Fig. 1b). For example, the primary degraders were *Acinetobacter, Gordonia*, *Devosia* and *Bdellovibrio* for n-octadecane in the C18 treatment; *Sphingobium, Sphingomonas, Novosphingobium* and *Naxibacter* for phenanthrene in the PHE treatment; *Hydrogenophaga* for the mixture of phenanthrene and n-octadecane in the PC treatment; and *Delftia*, *Dokdonella*, *Pigmentiphaga*, and *Aquabacterium* for the mixture of phenanthrene and n-octadecane in the presence of CdCl_2_ (PCC treatment).

The variation described above indicates that the differences in the microcosms (pollutants and the combination of pollutants) resulted in strong selection for the microbes in the consortia, consistent with the results of a previous study^30^. Significant effects of the different pollutants on microbial taxonomic dissimilarity (Bray–Curtis) and genetic diversity (Weighted UniFrac) were also revealed by the statistical analysis. These results likely suggest that organic pollutants, such as nutrient and heavy metal (CdCl_2_), function as environmental stresses for the selection of microorganisms^30^,^31^. However, the different compositions of decomposing bacteria between the PC treatment and the single-contaminant (C18 or PHE) treatments merit further study. In the final consortium of PC, the main decomposers were not a combination of those in C18 and PHE, demonstrating that the pollutants n-octadecane and phenanthrene not only functioned as substrates for degraders, but also as environmental factors for the selection of soil bacteria. That is, the presence of both n-octadecane and phenanthrene resulted in the simultaneous selection of degrader bacteria and toxic effects on the bacteria that are unable to degrade the compound. Thus, the microbial community for degradation the mixed pollutants is not simple as the combination of single compound degraders. A related study reported that the types of petroleum mixture can select the microbial population in soil environments^32^. Moreover, mixed contamination with heavy metals and PAHs can influence the microbial structure and function in soil^30^.

Secondly, the results clearly demonstrated that different pollutants and their combinations influenced the succession of bacterial community in distinct directions. To understand the direction and rate of the processes catalyzed by environmental microbial communities, we monitored the microbial diversity in the present study^33^. The decrease in richness, diversity and evenness of the microbial community during enrichment subcultures indicated that the consortia gradually matured. The gradual increase in the dissimilarities of the consortia composition among the different microcosms also confirmed this conclusion (Fig. 3, also Supplementary Fig. S6). The high complexity of the microbial communities presented in the initial stages of subcultures might reflect the intrinsically high and stochastic microbial influx from the initial soil^34^. These results are consistent with those of a previous study demonstrating that microorganisms with a potentially high growth rate are selected and become dominant with a consequent reduction in the evenness of the species distribution during batch-enrichment culture^35^.

Temporal variations based on taxonomic and phylogenetic beta-diversity, an often overlooked crucial aspect of microbial communities, can be elucidated based on a succession framework^34^. As succession proceeds, some microorganisms with low abundance in the initial phases gradually become dominant, whereas microbes that were initially predominant become depleted (Fig. 3). These changes might reflect the selection of pollutants as carbon/energy source and environmental stresses, similar to the progressive increase observe during composting; this selection is likely associated with the biochemical evolution of the microcosms in terms of growth-supporting substrates and niches^36^. In oil-polluted soil, fertilizer induces clear changes in the bacterial communities^37^. Pollutants trigger the directional succession of bacterial communities during the early stages of oil pollution, including in planted soils^38^. In addition, some studies have demonstrated that multiple biostimulation can remarkably alter the bacterial community^18^. The results of the present study suggest that a single environmental factor such as added nutrients or selection pressures, can significantly alter the microbial community structure and succession, potentially explaining the large differences in microbial community structures observed in diverse environments.

Thirdly, the microbial community structure and its succession were the result not only of interactions between microorganisms and environmental factors (pollutant compounds and presence of CdCl_2_), but also interactions among the microorganisms in the community. The more complex and compact network in the PHE consortia compared with the C18 consortia indicates that the members responding to phenanthrene degradation were more strongly correlated with each other and that the microbial community was more stable. Because phenanthrene is more difficult to utilize than n-octadecane, the cooperations among the microbes in the consortia might be more important for phenanthrene degradation. Accordingly, the PCC network was more complex and compact than the PC network. In addition, less cooperation among the microbes was observed in the PC and PCC consortia than in the PHE consortia. It could be explained that in the PC and PCC treatments, more types of microorganisms were enriched in the presence of the easily utilized n-octadecane compared with phenanthrene alone. Therefore, the strength of the co-operation in the microbial community increases with the increasing stress applied. Network topology not only provides an overview of the patterns of co-occurrence of microbial taxa within a given ecosystem, but also reveals the effect of these patterns on ecosystem properties^39^. For microorganisms, environmental filtering predicts the specific habitat limits and is affected by abiotic factors that can support the coexistence of species within the communities^40^. The comparison of network topology properties between different environments or ecosystems can reveal effect of environment on the microbial community assembly.

Fourthly, some genera were recorded for the first time as bacteria associated with the degradation of petroleum hydrocarbon in the present study. Numerous studies of the isolation and identification of pollutant-degraders have been conducted. Many of the bacteria playing key roles in n-octadecane degradation belong to *Exiguobacterium*, *Burkholderia*, *Bacillus*, and *Pseudomonas*, and phenanthrene-degraders within the genera *Pseudomonas*, *Burkholderia*, *Rhodococcus*, and *Acinetobacter* have also been reported^41^. Except the observation of above mentioned degraders, many other bacteria such as *Bosea*, *Simplicispira*, *Pigmentiphaga*, *Algoriphagus* etc. may also participated the degradation of employed pollutants, as degraders or by co-metabolism.

At the genus level, *Acinetobacter*, *Gordonia*, *Castellaniella* and *Sphingobium* might be n-octadecane degraders and were present in >5% of relative abundance at the final phase in the C18 consortia (Supplementary Table S4). These microbes have also been reported as alkane degraders in previous studies^11^,^42^,^43^. However, the detection of *Niabella* with a relative abundance of 6.84% in our C18 consortia, as well as in other three treatments, indicated it a novel member associated with the phenanthrene degradation. The genera *Sphingobium*, *Sphingopyxis*, *Castellaniella*, *Terrimonas*, and *Hydrogenophaga* might be the main phenanthrene-degraders, accounting for 90.27% of the total reads in the PHE enriched consortia (Supplementary Table S4). *Sphingobium* has been reported as common degraders of PAHs^44^ with the ability to degrade crude oil, diesel, and kerosene in crude oil-contaminated seashore^45^. *Terrimonas* and *Hydrogenophaga* were previously reported in PAH-degrading microbial communities^46^. *Castellaniella* degrades alkanes^43^ but has not been implicated in the degradation of PAHs.

When the CdCl_2_ was present, the genera *Delftia, Sphingobium, Dokdonella, Hydrogenophaga, Stenotrophomonas, Pseudoxanthomonas, Acinetobacter, Naxibacter, Chryseobacterium,* and *Nocardia* were predominant. These microorganisms might exhibit both hydrocarbon-degradation and metal-tolerant, but many of these microbes have been reported as bacteria associated with hydrocarbon degradation, and not metal tolerance. *Delftia*, *Stenotrophomonas* and *Pseudoxanthomonas* have been identified as PAH degraders^12^,^47^. *Dokdonella* was detected previously in the PAH-degrading consortia enriched from tsunami sediments^14^. *Naxibacter* exhibits high arsenic resistance^48^, and was initially identified in a hydrocarbon-degrading consortium.

To our knowledge, this study is the first to systematically depict the community structure of enriched microbial consortia degrading different pollutants, even in the presence of heavy metals, via high-throughput sequencing of the 16S rRNA gene. In the present study, four treatments finally induced four pollutants-degrading consortia through enrichment, and their richness, diversity and evenness of these populations decreased in successive subcultures. These consortia predominantly comprised known petroleum hydrocarbon degraders, such as *Acinetobacter*, *Gordonia*, *Sphingobium*, *Sphingopyxis* and *Castellaniella*. Several genera were novel record as bacteria associated with the degradation of petroleum hydrocarbon, such as *Niabella* and *Naxibacter*. In addition, the primary degrading groups differed among consortia responding to different pollutants. A few predominant microbial members were shared among the different treatments, even when the same carbon source was supplied in some cases. The results of the present study also indicate that different pollutants influence the direction of microbial succession, resulting in distinct consortia from identical initial microbial community. Moreover, comparing the properties of networks between treatments revealed that pollutants affect the microbial co-occurrence patterns. Overall, these results suggest that single environmental factors such as added nutrients or selection pressures, can significantly alter the microbial community structure. Thus, multiple factors should be considered in bioremediation practices.

## Methods

### Sampling sites

The contaminated surface soil sample (0–30 cm depth) used in the present study was collected around an oil refinery (E 108°46′09″ and N 34°21′35″) in Xianyang City, Northwest of China. This site has been continuously polluted with the discharged wastewater for more than 25 years and the concentration of total petroleum hydrocarbons (TPH) was 2.64 ± 0.01 g kg^−1^ of dry soil. The soil sample was stored at 4 °C in a sealed plastic bag until further use. To prepare the inoculum, 20 g of fresh soil was suspended in 180 ml of 0.85% NaCl solution. After agitation for 10 min at 240 rpm, an aliquot of 20 ml of supernatant was subsequently transferred to a 500-ml flask containing 180 ml of basal salt medium (BSM) and pollutants.

### Enrichment Cultures

The BSM used in the present study contained 4 g of K_2_HPO_4_, 4 g of Na_2_HPO_4_, 2 g of (NH_4_)_2_SO_4_, 0.2 g of MgSO_4_·7H_2_O, 0.001 g of CaCl_2_·2H_2_O, and 0.001 g of FeSO_4_·7H_2_O in 1 l of distilled water, pH 7, adjusted with 1 N H_2_SO_4_. After autoclaving at 121 °C for 15 min, the BSM was supplemented with one of the following: (1) 500 mg l^−1^ of phenanthrene (PHE), (2) 500 mg l^−1^ of n-octadecane (C18), (3) 250 mg l^−1^ of phenanthrene + 250 mg l^−1^ of n-octadecane (PC), and (4) 250 mg l^−1^ of phenanthrene + 250 mg l^−1^ of n-octadecane + 50 mg l^−1^ of CdCl_2_ (PCC). Briefly, the organic materials dissolved in dichloromethane were added to empty flasks and the solvent was evaporated; BSM was then added and the mixture was inoculated with a soil suspension (inoculum). Flasks were prepared incubated in triplicate for each treatment and incubated with shaking at 150 rpm in the dark at 28 ± 2 °C; flasks without inoculum were prepared as blank controls. Ten successive subcultures were prepared by transferring 10% (v/v) of the culture to a subsequent subculture in 10-day intervals for a total treatment period of 100 days. Cells from the consortium of each subculture were collected after centrifuging at 10,000 ×g for 15 min at room temperature for DNA extraction. The residual organic materials were extracted from the supernatant in an equal-volume of dichloromethane, and the concentration was determined by gas chromatography with flame ionization detection (GC-FID) (*method in* Supplementary Information). The biodegradation percentage was calculated as the difference in the concentrations of the pollutants between the blank control and the treatments.

### DNA preparation and MiSeq sequencing

Metagenomic DNA was extracted from the initial soil sample and each of the enriched microbial consortia using the MP FastDNA®SPIN Kit for soil (MP Biochemicals, Solon, OH, USA) and the SDS-CTAB method, respectively. The V4–V5 hypervariable region of the 16S rRNA gene was amplified using the primers 515F (5′-GTG CCA GCM GCC GCG GTA A-3′) and 926R (5′-CCG YCA ATT YMT TTR AGT TT-3′) with a sample tagging approach. These primers are complementary to the Illumina forward, reverse, and multiplex sequencing primers (with the reverse primer also contains a 12-bp index to permit multiplexing). Amplification was conducted using a PCR thermal cycler Model C1000 (Bio-Rad, Richmond, CA). The total volume of the reaction mixture was 50 μl and included 0.5 μl of each primer (50 pmol each), 5 μl of 2.5 mmol l^−1^ dNTP mixture, 5 μl of 10 × ExTaq buffer (20 mmol l^−1^ Mg^2+^; TaKaRa Inc., Dalian, China), 0.25 μl of ExTaq DNA polymerase (TaKaRa), 1 μl of the sample DNA template and 37.75 μl of Milli-Q water. The cycle conditions included initial denaturation at 94 °C for 3 min, followed by 30 cycles of denaturation at 94 °C for 30 s, annealing at 50 °C for 30 s and extension at 72 °C for 30 s, and an extension step at 72 °C for 5 min after cycling was complete. All samples were amplified in triplicate, and no-template controls were included in all steps of the process. Next, 5 μl of each reaction mixture was analyzed by electrophoresis on a 2% (w/v) agarose gel, and the PCR products were gel-purified using a QIAquick Gel Extraction Kit (Qiagen, Hilden, Germany). The concentrations of the amplicons were determined using the Quant-iT PicoGreen dsDNA reagent kit (Life Technologies, Merelbeke, Belgium), and the purified amplicons from each consortium were combined in equimolar ratios. The amplicons were subsequently vacuum dried and sequenced using an Illumina MiSeq (250-bp paired-end reads) platform at Macrogen Inc. (http://www.macrogen.com, Seoul, South Korea).

### Sequence analysis of the 16S rRNA amplicons

The reads from each of the DNA samples were merged using FLASH (V1.2.7, http://ccb.jhu.edu/software/FLASH/), and quality filtering of reads was performed as previously described^49^. The acquired sequences were denoised (homopolymer error-correction) using Denoiser V0.91 software according to the manual. Chimeric sequences were removed using the USEARCH software based on the UCHIME algorithm^50^. The sequences were subsequently assigned to each sample with a 12-bp barcode using a script derived from the QIIME pipeline. The remaining sequences from all samples were clustered into Operational Taxonomic Units (OTUs) at 97% sequence similarity using an “uclust” model (search and clustering orders of magnitude faster than BLAST). The representative sequences for each OTU were assigned to taxonomic groups using the RDP classifier at an 80% confidence threshold.

### Data analyses

Prior to data analysis, a subsample of a minimum number of sequences (15,000) from each sample was used to remove all potential side effects of the sample size. For alpha diversity analysis, Chao1 richness, OTU richness, Pielou’s evenness and Shannon index were calculated with 3 iterations using a step size of 100 sequences per sample. To identify connections between the treatments and the microbial patterns, the beta diversity between the enriched consortia was estimated based on the pairwise Weighted UniFrac and Bray-Curtis dissimilarity distances with QIIME (http://qiime.org/index.html). PCoA was performed on the distance matrices to visualize the sample relationships.

ANOSIM^51^ and permutational multivariate analysis of variance (ADONIS)^52^ were performed to determine whether sample classifications (different treatments) contained significant differences in phylogenetic or species diversity based on Weighted UniFrac and Bray-Curtis distance matrices. Significant taxonomic differences between treatments were tested using LDA effect size (Lefse)^53^. We employed the factorial Kruskal-Wallis sum-rank test (α = 0.05) to identify taxa with significant differential abundances between treatments (using one-against-all comparisons), followed by LDA (LDA >2) to estimate the effect size of each differentially abundant feature. The resulting significant taxa were used to generate a taxonomic cladogram illustrating differences between treatments.

Together, Random forest (RF) supervised-classification models^54^ were used to identify taxonomic features explaining the strongest variation between treatments and evaluate the diagnostic strength of these features to discriminate against classifications. Using taxonomic assignments of OTUs as predictors and treatments as class labels, models were constructed with an optimal number 2000 trees; the OOB errors did not decrease with increasing tree number, that evidence the appropriateness of the RF supervised-classification models in this study. RF provides a measure to estimate the importance of features (taxa) based on the mean decrease in classification accuracy with permutation.

The network was used to explore co-occurrence patterns of microbial taxa. The consortia from ten stages of each treatment were grouped to generate a network. The genera with relative abundances greater than 0.05% were selected. A Spearman’s correlation between two genera was considered statistically robust when the Spearman’s correlation coefficient (ρ) was >0.6 and the *P*-value was <0.01^19^. All robust correlations identified from pairwise comparison of the genera abundance formed a correlation network in which each node represented one genus, and each edge represented a strong and significant correlation between the nodes. To describe the topology of the resulting networks, a set of measures (number of nodes and edges, average path length, network diameter, average degree, graph density, clustering coefficient and modularity) was calculated and networks were visualized using the interactive platform Gephi^55^.

All statistical analyses were performed in the R environment (http://www.r-project.org) using vegan^56^, igraph^57^ and Hmisc^58^ packages.

## Additional Information

**How to cite this article**: Jiao, S. *et al.* Microbial succession in response to pollutants in batch-enrichment culture. *Sci. Rep.* **6**, 21791; doi: 10.1038/srep21791 (2016).

## Supplementary Material

Supplementary Information

Supplementary Datasets 1

## Figures and Tables

**Table 1:**
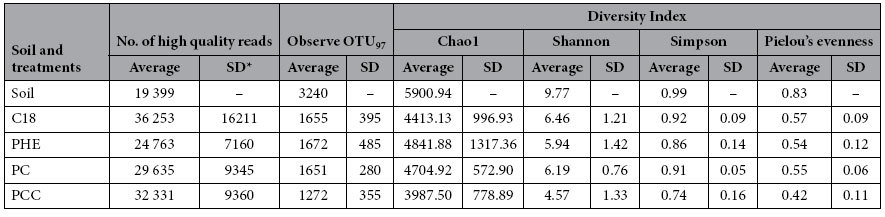
Microbial alpha-diversity characteristics for enriched consortia and original soil.

^*^standard deviation.

**Table 2:** ANOSIM and permutational MANOVA of microbial diversity among different treatments in three phases.

Phases	Bray–Curtis				weighted UniFrac			
	ANOSIM		ADONIS		ANOSIM		ADONIS	
	R	*P*	R^2^	*P*	R	*P*	R^2^	*P*
I	0.068	0.297	0.278	0.412	−0.028	0.576	0.287	0.341
II	0.722	0.001	0.490	0.001	0.677	0.001	0.578	0.001
III	0.951	0.001	0.760	0.001	0.889	0.001	0.819	0.002

**Table 3:** Topological properties of co-occurring networks among different treatments.

Treatment	Node	Edge	Modularity	Clustering coefficient	Average path length	Network diameter	Graph density	Average degree
C18	84	119	0.776	0.400	5.620	10	0.034	2.833
PHE	74	513	0.335	0.582	2.695	6	0.190	13.865
PC	56	95	0.753	0.469	4.119	9	0.062	3.393
PCC	55	203	0.429	0.540	2.762	6	0.137	7.382
