# Supplementary material for: Microbial succession in response to pollutants in batch-enrichment culture
Source: Sci Rep. 2016 Feb 24;6:21791. doi: 10.1038/srep21791 (PMC4764846; doi:10.1038/srep21791)
Supplement: Supplementary Information [file srep21791-s1.pdf]

Supplementary materials for

Microbial successions responding to pollutants in batch-enrichment culture

Shuo Jiao[1], Weimin Chen[1], Entao Wang[2], Junman Wang[1], Zhenshan Liu[1], Yining Li[1], Gehong Wei[1*]

Analysis of residual organics by gas chromatography

The residual organics was analyzed by gas chromatography (GC) using a FID detector (SHIMADZU, GC-2010 plus). The column was SE30 capillary column (30 m long, 0.25 mm inside diameter, 0.25 mm film thickness) with helium as a carrier gas. The initial oven temperature was 80 ℃ and was heated at a rate of 50 ℃ min^{-1} to 200 ℃ holding for 1 min, then at a rate of 1 ℃ min^{-1} to 210 ℃. The injector temperature was 290 ℃ and the detector temperature was 290 ℃. The total helium (He) flow rate was 30.0 ml min^{-1}, the column flow rate was 30.0 ml min^{-1}. Individual components were determined by matching the retention time with authentic standards.

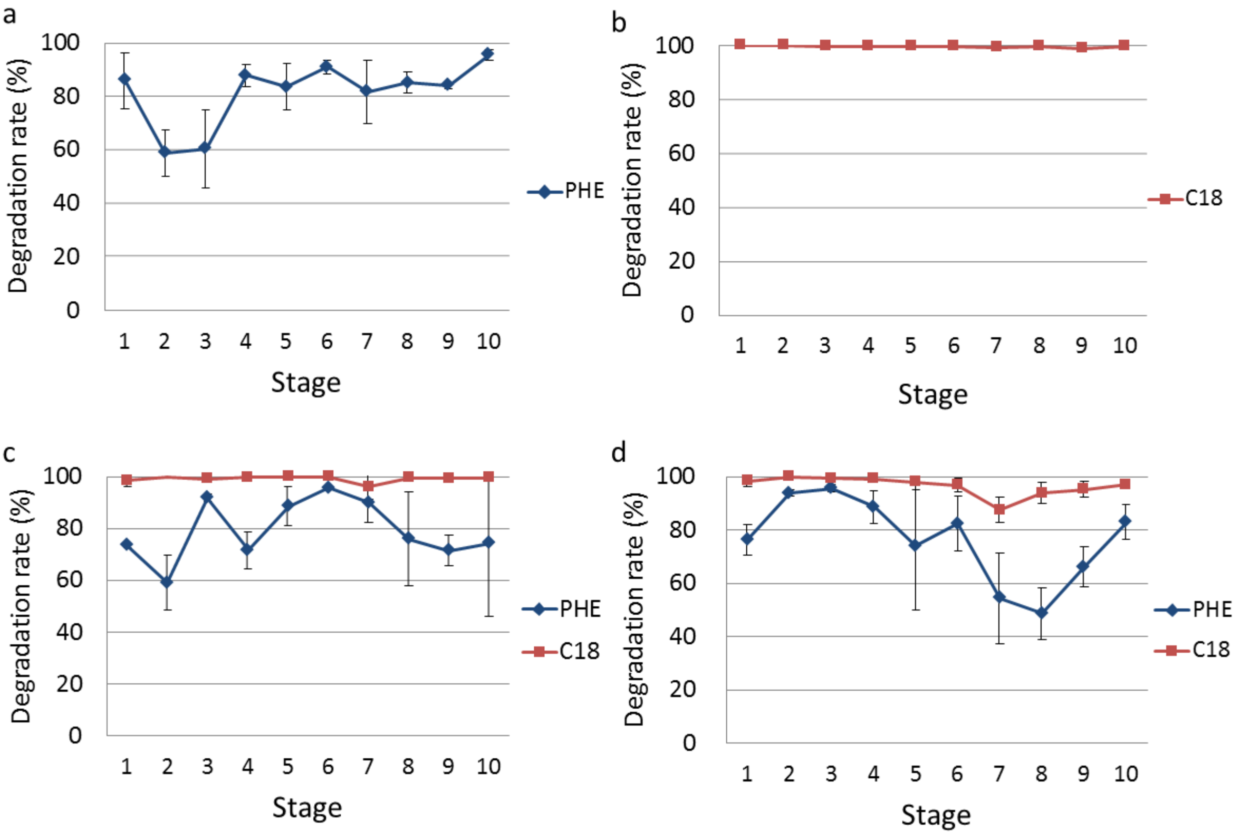

Supplementary Fig. S1. Degradation rate of n-octadecane and phenanthrene in the four treatments at ten stages. (a) PHE: with 500 mg l^{-1} phenanthrene; (b) C18: with 500 mg l^{-1} n-octadecane alone; (c) PC: 250 mg l^{-1} of phenanthrene + 250 mg l^{-1} of n-octadecane; (d) PCC: 250 mg l^{-1} of phenanthrene + 250 mg l^{-1} of n-octadecane + 50 mg l^{-1} of $CdCl_2$. PHE and C18 in the graph refer to phenanthrene and n-octadecane, respectively.

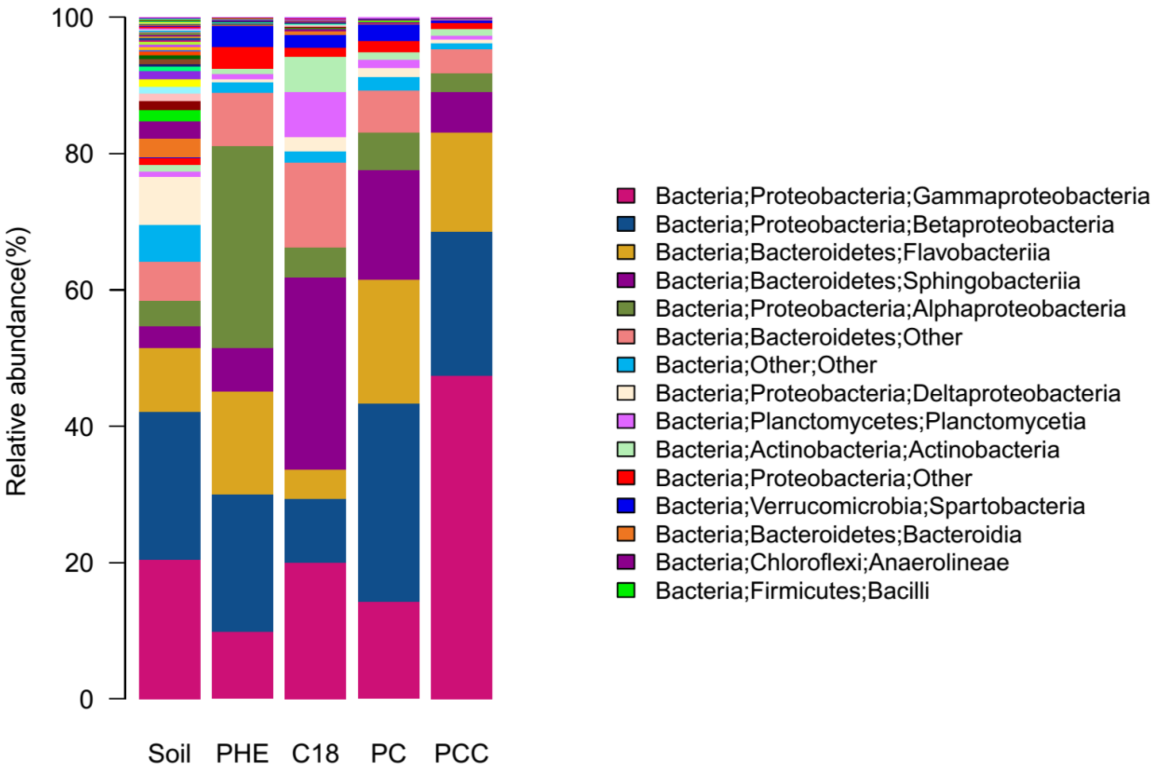

Supplementary Fig. S2. Average relative abundances of classes in the consortia of the different treatments and the original soil. “Soil” in the graph refer to the original soil.

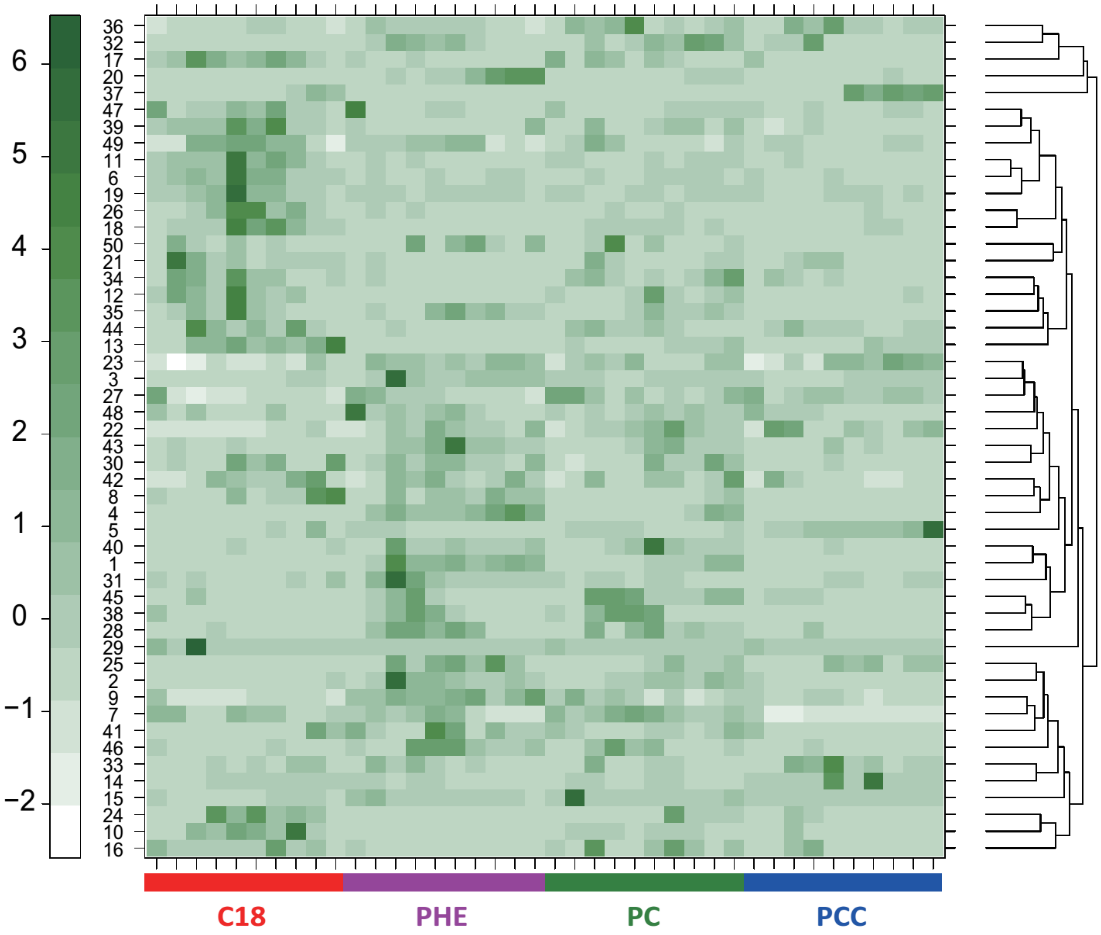

Supplementary Fig. S3. Hierarchical clustering and heatmap representation based on relative abundance of the first 50 important taxonomic features for treatments prediction in the Random Forest classification model. The treatments are presented on the horizontal axis, and the vertical axis represents the standardized values for taxonomic features (taxa). Taxonomic features were clustered based on average linkage and uncentered correlation similarity metric. Each column in the heatmap has been standardized to have a mean of zero and a standard deviation of one. The intensity of the green color in the heatmap is proportional to the standardized relative abundance of the taxa. The first 50 important taxonomic features are presented in Table S3.

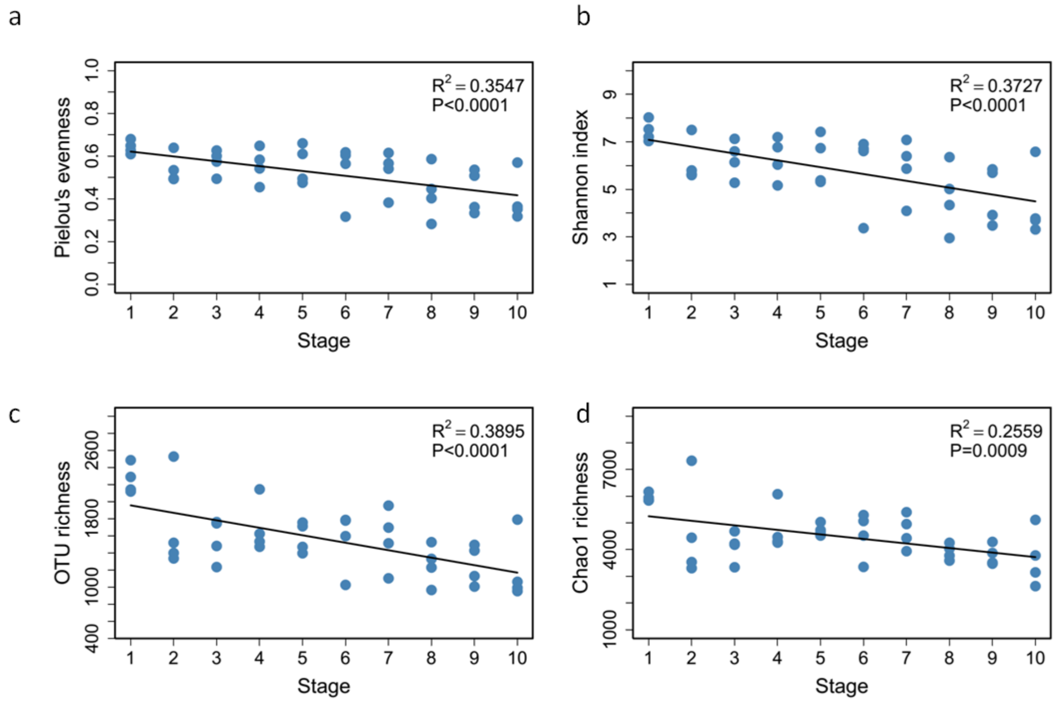

Supplementary Fig. S4. Changes in alpha diversity measurements change across the 10 stages. Linear regression of Pielou's evenness (a), Shannon index (b), OTU richness (c) and Chao1 richness (d).

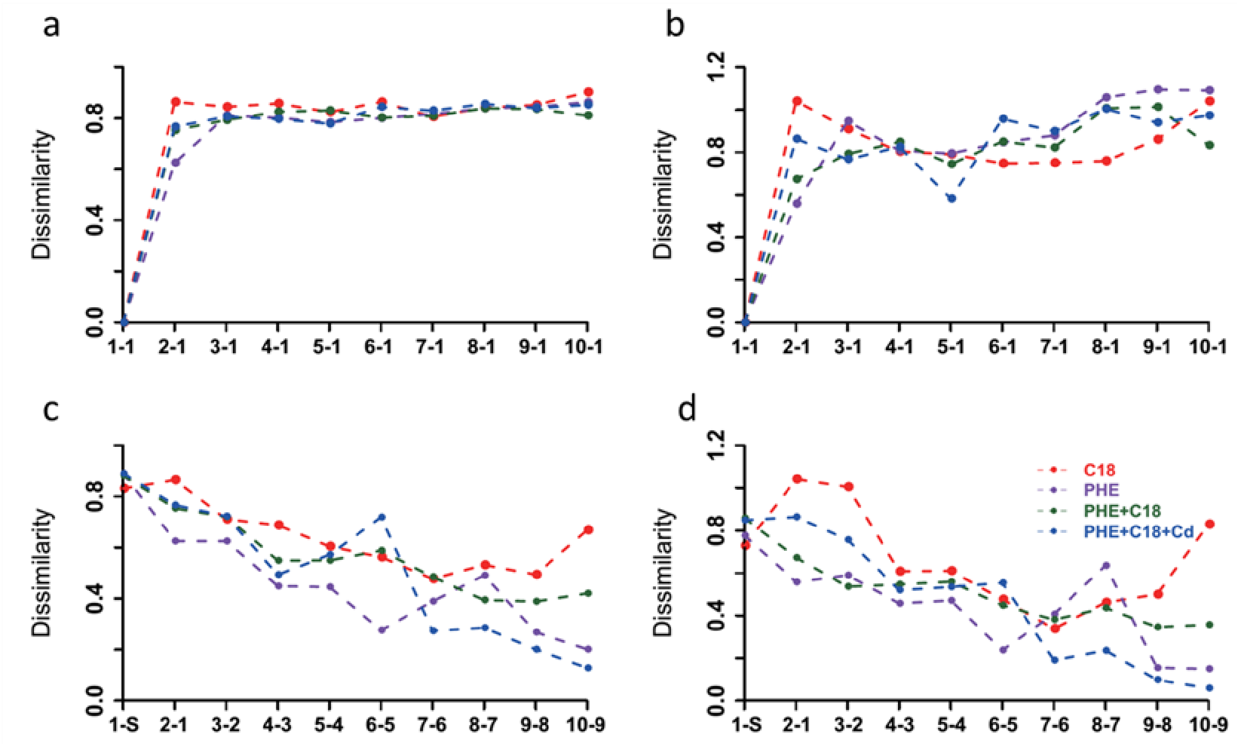

Supplementary Fig. S5. Community composition dissimilarity within treatments. Weighted UniFrac (a) and Bray-Curtis (b) distance between the first stage and other stages (i.e., comparing stage 1 with stage 2, 1-2; stage 1 with stage 3, 1-3, etc.; the graph begins at 100% for stage 1 vs. stage 1). Weighted UniFrac (c) and Bray-Curtis (d) distance between two adjacent stages (i.e., comparing initial soil with stage 1, 1-S; stage 1 with stage 2, 2-1; stage 2 with stage 3, 3-2, .etc.; the graph begins at 100% for stage 1 vs. initial soil).

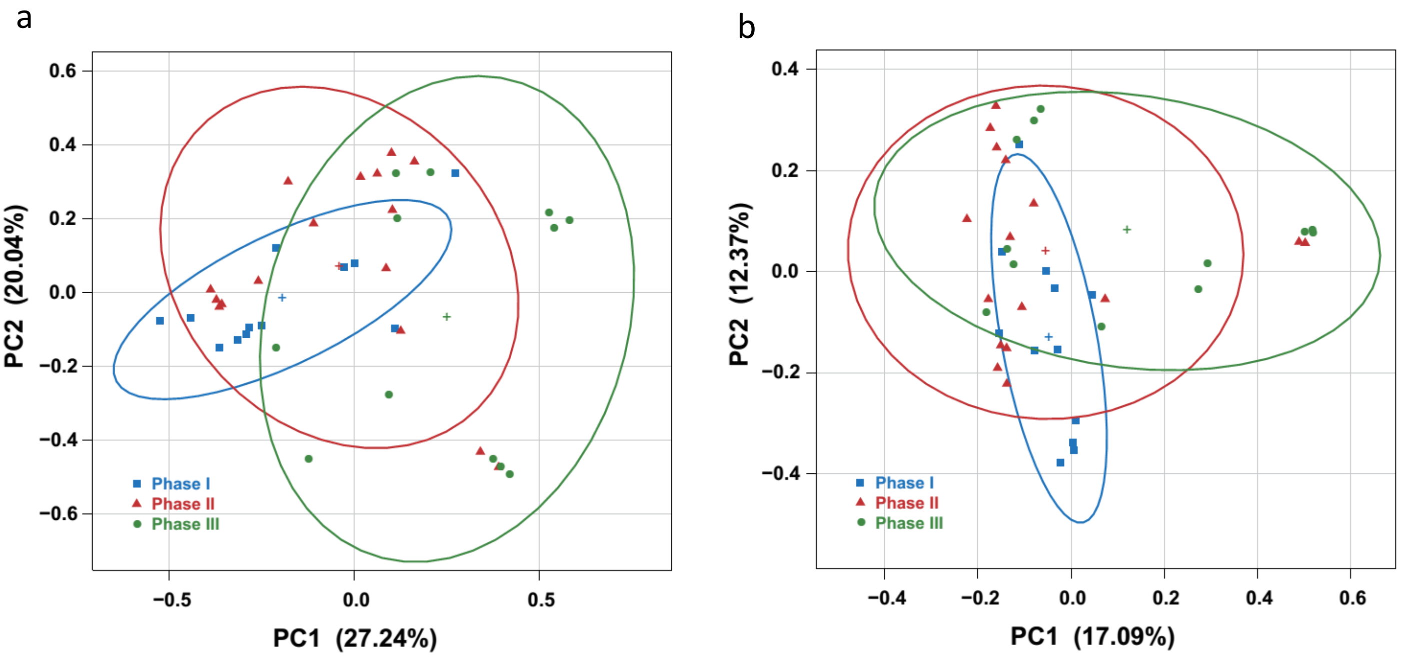

Supplementary Fig. S6. Microbial community dissimilarity among phases based on (a) Weighted UniFrac and (b) Bray-Curtis distance principal coordinate analysis (PCoA) of microbial communities; 80% confidence ellipses are shown around each phase group.

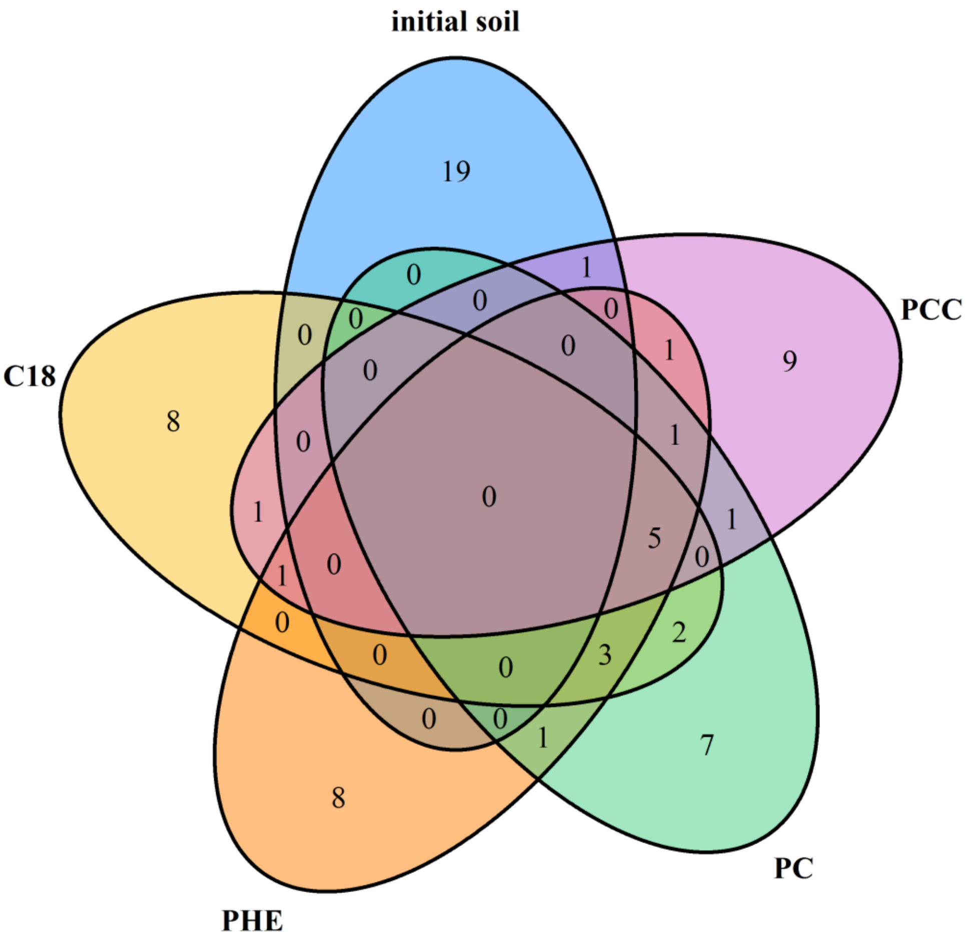

Supplementary Fig. S7. Venn diagram of the relative abundances of first 20 OTUs in the initial soil and the enriched consortia at phases III.

Supplementary Table S1. Degradation rate of different treatment across the 10 stages

Treatment*	Removed pollutant	Degradation rate at the subculture stage (%)										Average of the 10 stages (%)
		1	2	3	4	5	6	7	8	9	10	
PHE	Phe	85.98	58.93	60.46	87.84	83.54	91.01	81.64	85.16	83.99	95.49	81.40
C18	C18	99.98	100.0	99.83	99.79	99.60	99.82	99.24	99.82	98.85	99.87	99.68
PC	Phe	73.54	59.05	92.02	71.70	88.61	95.77	90.11	76.11	71.59	74.36	79.21
PC	C18	98.60	100.0	99.24	99.80	100.0	100.0	96.27	99.68	99.50	99.59	99.27
PCC	Phe	76.41	94.01	95.80	88.72	74.19	82.51	54.58	29.54	66.19	83.04	74.50
PCC	C18	98.55	100.0	99.42	99.19	98.14	96.93	87.56	94.01	95.34	97.06	96.62

*. PHE: with 500 phenanthrene (phe) alone; C18: with 500 mg l^{-1} n-octadecane (C18) alone; PC: mixture of 250 mg l^{-1} of each of Phe and C18; PCC: 250 mg l^{-1} of phenanthrene + 250 mg l^{-1} of n-octadecane + 50 mg l^{-1} of $CdCl_2$.

Supplementary Table S2. The distribution of different levels in each treatment and the original soil

Soil and treatments	The number of taxa in different level				
	Phylum	Class	Order	Family	Genus
Soil	32	70	106	192	358
C18	28	60	104	183	489
PHE	26	54	98	169	343
PC	26	52	91	161	319
PCC	22	48	84	154	311

Supplementary Table S3. Feature importance scores for Random Forest models

Rank	Feature (phylum; class; order; family; genus)	MDA	SD
1	Proteobacteria; Alphaproteobacteria; Sphingomonadales; Other; Other	0.02054	0.00238
2	Proteobacteria; Alphaproteobacteria; Sphingomonadales; Sphingomonadaceae; Sphingopyxis	0.02011	0.00159
3	Proteobacteria; Alphaproteobacteria; Sphingomonadales; Sphingomonadaceae; Novosphingobium	0.01998	0.00271
4	Proteobacteria; Alphaproteobacteria; Sphingomonadales; Sphingomonadaceae; Other	0.01692	0.00214
5	Proteobacteria; Betaproteobacteria; Burkholderiales; Comamonadaceae; Delftia	0.01687	0.00092
6	Proteobacteria; Alphaproteobacteria; Rhizobiales; Hyphomicrobiaceae; Devosia	0.01664	0.00084
7	Other; Other; Other; Other; Other	0.01618	0.00086
8	Proteobacteria; Alphaproteobacteria; Other; Other; Other	0.01301	0.00174
9	Proteobacteria; Betaproteobacteria; Burkholderiales; Other; Other	0.01296	0.00205
10	Actinobacteria; Actinobacteria; Actinomycetales; Nocardiaceae; Gordonia	0.01252	0.00161
11	Actinobacteria; Actinobacteria; Actinomycetales; Other; Other	0.01137	0.00076
12	Actinobacteria; Actinobacteria; Actinomycetales; Microbacteriaceae; Leucobacter	0.01127	0.00125
13	Proteobacteria; Alphaproteobacteria; Rhizobiales; Phyllobacteriaceae; Aquamicrobium	0.00911	0.00093
14	Proteobacteria; Gammaproteobacteria; Xanthomonadales; Xanthomonadaceae; Dokdonella	0.00838	0.00098
15	Proteobacteria; Betaproteobacteria; Burkholderiales; Comamonadaceae; Hydrogenophaga	0.00802	0.00081
16	Proteobacteria; Deltaproteobacteria; Myxococcales; Polyangiaceae; Byssovorax	0.00770	0.00131
17	Bacteroidetes; Sphingobacteriia; Sphingobacteriales; Chitinophagaceae; Other	0.00521	0.00075
18	Actinobacteria; Actinobacteria; Actinomycetales; Nakamurellaceae; Nakamurella	0.00514	0.00099
19	Proteobacteria; Deltaproteobacteria; Bdellovibrionales; Bdellovibrionaceae; Bdellovibrio	0.00501	0.00052
20	Proteobacteria; Alphaproteobacteria; Sphingomonadales; Sphingomonadaceae; Sphingobium	0.00431	0.00111
21	Actinobacteria; Actinobacteria; Actinomycetales; Microbacteriaceae; Microbacterium	0.00404	0.00104
22	Proteobacteria; Betaproteobacteria; Burkholderiales; Alcaligenaceae; Pigmentiphaga	0.00357	0.00051
23	Proteobacteria; Betaproteobacteria; Burkholderiales; Oxalobacteraceae; Naxibacter	0.00348	0.00095
24	Bacteroidetes; Sphingobacteriia; Sphingobacteriales; Chitinophagaceae; Niabella	0.00321	0.00095
25	Proteobacteria; Betaproteobacteria; Burkholderiales; Alcaligenaceae; Other	0.00306	0.00058
26	Actinobacteria; Actinobacteria; Actinomycetales; Nocardioidaceae; Aeromicrobium	0.00306	0.00054
27	Proteobacteria; Betaproteobacteria; Burkholderiales; Burkholderiales_incertae_sedis; Aquabacterium	0.00296	0.00059
28	Bacteroidetes; Flavobacteriia; Flavobacteriales; Flavobacteriaceae; Moheibacter	0.00286	0.00060
29	Bacteroidetes; Sphingobacteriia; Sphingobacteriales; Other; Other	0.00283	0.00055
30	Proteobacteria; Betaproteobacteria; Rhodocyclales; Rhodocyclaceae; Shinella	0.00264	0.00074
31	Proteobacteria; Alphaproteobacteria; Sphingomonadales; Sphingomonadaceae; Sphingomonas	0.00261	0.00091
32	Bacteroidetes; Flavobacteriia; Flavobacteriales; Flavobacteriaceae; Other	0.00259	0.00069
33	Proteobacteria; Gammaproteobacteria; Xanthomonadales; Xanthomonadaceae; Stenotrophomonas	0.00245	0.00050
34	Actinobacteria; Actinobacteria; Actinomycetales; Microbacteriaceae; Leifsonia	0.00219	0.00093
35	Proteobacteria; Alphaproteobacteria; Rhizobiales; Bradyrhizobiaceae; Nitrobacter	0.00218	0.00064
36	Proteobacteria; Betaproteobacteria; Burkholderiales; Comamonadaceae; Other	0.00213	0.00053
37	Proteobacteria; Gammaproteobacteria; Pseudomonadales; Pseudomonadaceae; Other	0.00211	0.00056
38	Proteobacteria; Alphaproteobacteria; Rhodospirillales; Acetobacteraceae; Other	0.00208	0.00045
39	Proteobacteria; Deltaproteobacteria; Myxococcales; Polyangiaceae; Other	0.00208	0.00042
40	Proteobacteria; Alphaproteobacteria; Rhizobiales; Brucellaceae; Ochrobactrum	0.00171	0.00056
41	Proteobacteria; Other; Other; Other; Other	0.00168	0.00060
42	Proteobacteria; Betaproteobacteria; Burkholderiales; Alcaligenaceae; Castellaniella	0.00156	0.00063

43	Proteobacteria; Alphaproteobacteria; Rhizobiales; Bradyrhizobiaceae; Bosea	0.00148	0.00074
44	Actinobacteria; Actinobacteria; Actinomycetales; Nocardiaceae; Other	0.00137	0.00085
45	Proteobacteria; Betaproteobacteria; Burkholderiales; Comamonadaceae; Comamonas	0.00129	0.00042
46	Proteobacteria; Betaproteobacteria; Nitrosomonadales; Nitrosomonadaceae; Nitrosomonas	0.00128	0.00064
47	Proteobacteria; Deltaproteobacteria; Myxococcales; Other; Other	0.00125	0.00050
48	Bacteroidetes; Flavobacteriia; Flavobacteriales; Other; Other	0.00116	0.00041
49	Proteobacteria; Betaproteobacteria; Burkholderiales; Comamonadaceae; Simplicispira	0.00113	0.00050
50	Verrucomicrobia; Other; Other; Other; Other	0.00108	0.00059

MDA, Mean decrease in accuracy; SD, standard deviation.

Supplementary Table S4. Relative abundance of first 20 genera in enriched consortia at phases III

Treatment							
C18		PHE		PC		PCC	
Genus	%	Genus	%	Genus	%	Genus	%
Acinetobacter	14.99	*Sphingobium*	85.55	*Hydrogenophaga*	17.38	*Delftia*	10.47
Gordonia	12.03	*Sphingopyxis*	1.64	*Sphingobium*	12.73	*Sphingobium*	10.45
Niabella	6.84	*Castellaniella*	1.27	*Sphingopyxis*	11.00	*Dokdonella*	9.38
Castellaniella	5.25	*Terrimonas*	0.91	*Legionella*	9.67	*Hydrogenophaga*	8.95
Sphingobium	4.68	*Hydrogenophaga*	0.89	*Niabella*	4.87	*Stenotrophomonas*	7.04
Shinella	4.37	*Acinetobacter*	0.83	*Terrimonas*	4.30	*Pseudoxanthomonas*	5.57
Stenotrophomonas	4.32	*Nitrosomonas*	0.82	*Castellaniella*	4.23	*Acinetobacter*	5.02
Hydrogenophaga	3.28	*Naxibacter*	0.55	*Byssovorax*	3.16	*Naxibacter*	4.01
Dokdonella	2.93	*Novosphingobium*	0.53	*Acinetobacter*	2.76	*Chryseobacterium*	2.98
Azospira	2.92	*Pseudoxanthomonas*	0.52	*Shinella*	2.27	*Nocardia*	2.54
Terrimonas	2.35	*Shinella*	0.51	*Stenotrophomonas*	2.00	*Terrimonas*	2.18
Chryseobacterium	2.29	*Bosea*	0.49	*Pseudoxanthomonas*	1.98	*Aquabacterium*	2.03
Sphingopyxis	2.13	*Chryseobacterium*	0.37	*Gordonia*	1.56	*Thermomonas*	1.81
Pseudoxanthomonas	2.07	*Parvibaculum*	0.32	*Naxibacter*	1.43	*Diaphorobacter*	1.74
Proteiniphilum	1.68	*Thermomonas*	0.32	*Chryseobacterium*	1.40	*Novosphingobium*	1.61
Byssovorax	1.66	*Legionella*	0.31	*Parvibaculum*	1.27	*Pigmentiphaga*	1.54
Naxibacter	1.53	*Dokdonella*	0.29	*Bosea*	1.25	*Sphingopyxis*	1.47
Sedimentibacter	1.38	*Stenotrophomonas*	0.27	*Nitrosomonas*	1.22	*Parvibaculum*	1.44
Thermomonas	1.27	*Pigmentiphaga*	0.25	*Dokdonella*	1.21	*Escherichia/Shigella*	1.33
Methylobacillus	1.16	*Ochrobactrum*	0.22	*Thermomonas*	0.98	*Gordonia*	1.31

%: relative abundance of the genera
